# Nutrient status changes bacterial interactions in a synthetic community

**DOI:** 10.1128/aem.01566-23

**Published:** 2023-12-21

**Authors:** Yizhu Qiao, Qiwei Huang, Hanyue Guo, Meijie Qi, He Zhang, Qicheng Xu, Qirong Shen, Ning Ling

**Affiliations:** 1 Key Lab of Organic-based Fertilizers of China, Jiangsu Provincial Key Lab for Solid Organic Waste Utilization, Nanjing Agricultural University, Nanjing, China; 2 Centre for Grassland Microbiome, State Key Laboratory of Grassland Agro Ecosystems, College of Pastoral Agriculture Science and Technology, Lanzhou University, Lanzhou, China; Georgia Institute of Technology, Atlanta, Georgia, USA

**Keywords:** microbial interactions, metabolic similarity, niche deformation, resource availability

## Abstract

Microbial interactions affect community stability and niche spaces in all ecosystems. However, it is not clear what factors influence these interactions, leading to changes in species fitness and ecological niches. Here, we utilized 16 monocultures and their corresponding pairwise co-cultures to measure niche changes among 16 cultivable bacterial species in a wide range of carbon sources, and we used resource availability as a parameter to alter the interactions of the synthetic bacterial community. Our results suggest that metabolic similarity drives niche deformation between bacterial species. We further found that resource limitation resulted in increased microbial inhibition and more negative interactions. At high resource availability, bacteria exhibited little inhibitory potential and stronger facilitation (in 71% of cases), promoting niche expansion. Overall, our results show that metabolic similarity induces different degrees of resource competition, altering pairwise interactions within the synthetic community and potentially modulating bacterial niches. This framework may lay the basis for understanding complex niche deformation and microbial interactions as modulated by metabolic similarity and resource availability.

**IMPORTANCE:**

Understanding the intricate dynamics of microbial interactions is crucial for unraveling the stability and ecological roles of diverse ecosystems. However, the factors driving these interactions, leading to shifts in species fitness and ecological niches, remain inadequately explored. We demonstrate that metabolic similarity serves as a key driver of niche deformation between bacterial species. Resource availability emerges as a pivotal parameter, affecting interactions within the community. Our findings reveal heightened microbial inhibition and more negative interactions under resource-limited conditions. The prevalent facilitation is observed under conditions of high resource availability, underscoring the potential for niche expansion in such contexts. These findings emphasize that metabolic similarity induces varying degrees of resource competition, thereby altering pairwise interactions within the synthetic community and potentially modulating bacterial niches. Our workflow has broad implications for understanding the roles of metabolic similarity and resource availability in microbial interactions and for designing synthetic microbial communities.

## INTRODUCTION

Microbes are widely acknowledged to play a major role in Earth’s biosphere through their participation in the decomposition and cycling of carbon, nitrogen, and sulfur, which have significant implications for the functioning of the ecosystem. Inhabiting a variety of environments, microbes do not exist in solitude (1) but rather form complex, highly diverse, and dynamic microbial communities in which members communicate, compete, and cooperate with each other (2, 3). Microbial community interactions are essential components of ecosystem function, exerting significant influence on vital processes, such as nutrient cycling (3), symbiotic relationships, biological control (4), and biogeochemical cycling (5). These interactions also play a pivotal role in shaping the composition, stability, and available niche space within microbial ecosystems (2, 3, 6). Microbial interactions are delineated into no interaction, competition, and cooperation based on changes in fitness and niche differentiation before and after the co-cultivation of two species (7). As such, the type of interactions that occur can alter the initial productivity and niche establishment of species. However, it remains unclear which factors influence these interactions, altering species fitness and ecological niches.

**Fig 1:**
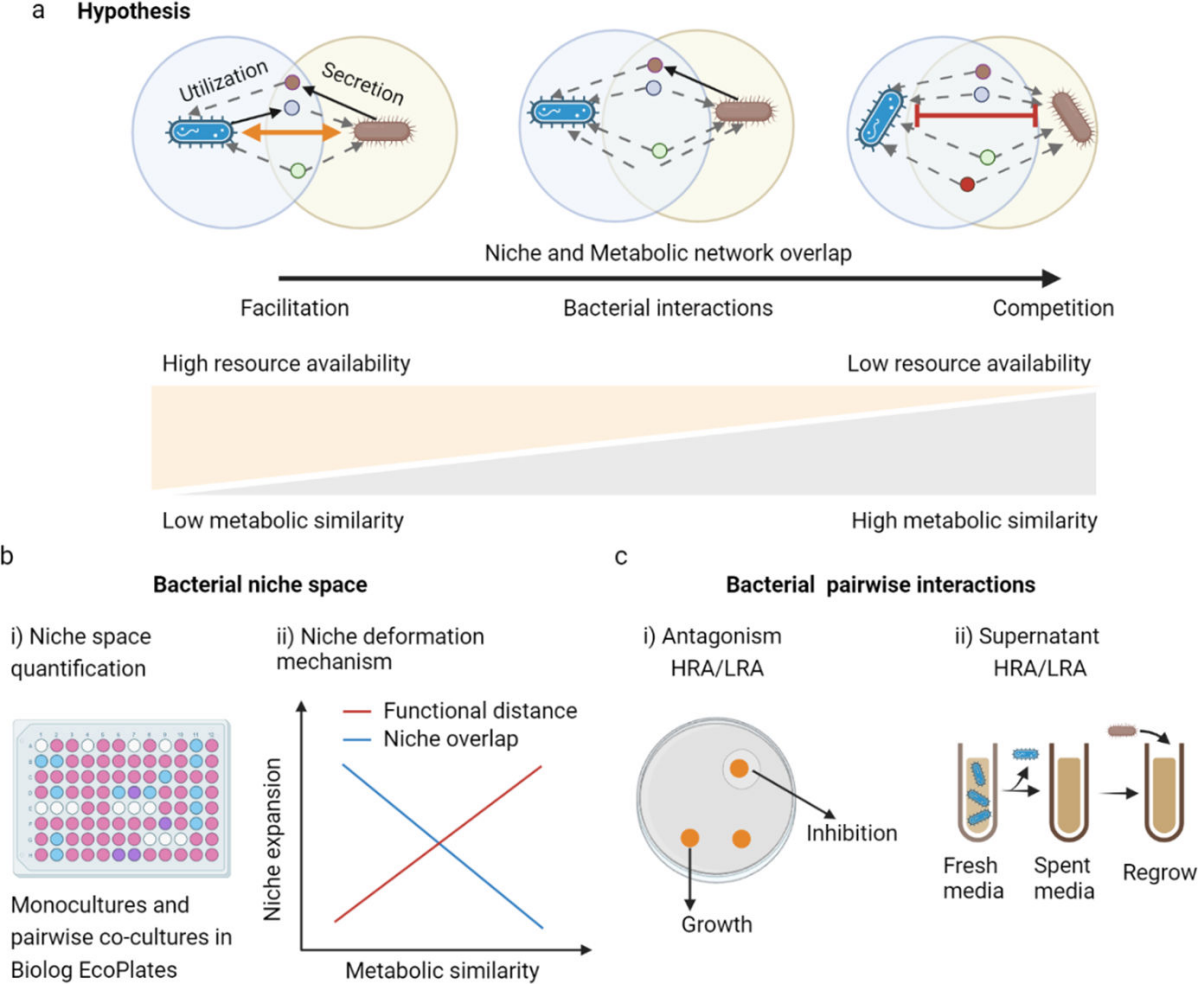
A schematic figure illustrating the hypothesis and experimental design. (a) Changes in microbial interactions due to resource availability as a potential access for niche deformation. (b) Quantification of niche space and deformation among bacteria. (c) Assessing pairwise interactions between bacteria through antagonism and supernatant assays, conducted under both low and high resource availability levels. LRA and HRA represent low resource availability and high resource availability, respectively.

Realized niche is generally considered to be the result of modification induced by the ecological interactions of species (8, 9). Although it is clear that cooperation and competition within microbial communities are central to their stability, maintenance, and niche deformation, the impact of each is manifested in various outcomes (6, 10). It has been hypothesized that congeneric species are usually more similar in phylogenetic and metabolic functions than species of different genera and that the competition for survival between them would be more intense (11). Studies have verified congeneric antagonism in bacteria and found that such antagonism is reciprocal, such as through the secretion of toxins (12), extracellular enzymes (13), and secondary metabolites (14). When the interfering species can disrupt other species at a low cost and there is a high overlap in resource utilization with the species being interfered against, the interference behavior is favored and strengthened. This allows the interfering species to gain a profit or to benefit by acquiring resources that other species would have used (15). Accordingly, antagonism should be most prevalent among metabolically similar species. In contrast, metabolically dissimilar species may have metabolites released into the environment as public goods or may carry out cross-feeding in favor of their partners because of differences in metabolic profiles (16, 17). A study on gut microbiomes emphasized the synergies in obligate cross-feeding, profoundly influencing the available niche space (8). Therefore, we hypothesize that with small metabolic similarity between species, their interactions may expand the ecological niche space through processes such as cooperative growth and individual niche reconstruction (Fig. 1a).

Changes in microbial interactions caused by metabolic similarity can be attributed to resource competition driven by resource availability (7, 8). In the context of the competition sensing hypothesis, in response to resource limitation, stress microbes upregulate the production of bacteriocins and antibiotics (18, 19), resulting in certain mutualisms becoming competitive (18). Bacteria have also been shown to be able to influence their surroundings through chemical modifications that directly affect their interactions with other community members (12, 17). Therefore, it is essential to connect species interactions to community dynamics in various complex communities (20, 21). In the highly fluctuating rhizosphere environment, where resource availability varies in space and time, the same microbial community can shift to become more cooperative or competitive, thus changing the ecological niche of the microbial community (19, 22). However, it is not clear how such interactions change with resource availability. Previous findings demonstrate that lower resource availability would allow bacterial populations to compete for limited public resources and would produce antimicrobial substances (19). This can prevent suppressed microorganisms from using certain resources, resulting in the loss of certain ecological niches. On the other hand, microbes have a higher fitness and produce larger amounts of metabolites under high resource availability (17, 23). In this condition, bacteria alter the surrounding chemical environment to increase resource availability by producing a greater diversity of secondary metabolites or public goods (24). Marsland and colleagues developed a consumer-resource model to study metabolite exchange based on community composition and metabolite fluxes (14). They revealed that a higher rate of external resource supply corresponded to an increased metabolite flux between community members, and the majority of biomass was predicted to originate from not only the primary resource but also the exchanged metabolites. Thus exchange of metabolites, like cross-feeding, may result more from a positive interaction (14, 24, 25). These positive interactions are pervasive in natural ecological communities and have been shown to play key roles in many critical ecosystem processes (16). For example, some studies have shown that these positive metabolic dependencies, or interactions, are a major driver of species co-occurrence (8, 23, 25–27). Accordingly, we hypothesized that low metabolic similarity and low resource competition (under higher resource availability) between strains would lead to stronger facilitative interactions (Fig. 1a).

To test these hypotheses, we utilized a synthetic bacterial community consisting of 16 isolates within 16 different genera collected from the rhizosphere of watermelon to quantify bacterial niche and pairwise interactions within the synthetic community and investigate how these interactions are altered by metabolic similarity (niche overlap and functional distance). We first quantified bacterial niche space (i.e., the number of carbon sources that a strain could utilize) and systematically compared the metabolic niche spaces of these strains in monocultures and corresponding paired co-cultures with 31 different carbon (C) sources after a period of cultivation (Fig. 1b). Furthermore, to gain a broad understanding of the changes in species interaction under resource competition, we conducted antagonistic experiments and supernatant assays under different resource availabilities to test all pairwise interactions and their variation trends between the strains (Fig. 1c). Overall, this work suggests that metabolic similarities and resource availability co-drive changes in the bacterial niche by altering bacterial interactions in our synthetic communities and that more cooperative relationships occur under high resource availability (low resource competition). More broadly, this work reveals that the modifications of niches and pairwise interactions within the synthetic community depend on metabolic characteristics and resource availability, which can pave the way for understanding basic interactions between microbes.

## RESULTS

### Pairwise interactions influence niche deformation between bacterial species

**Fig 2:**
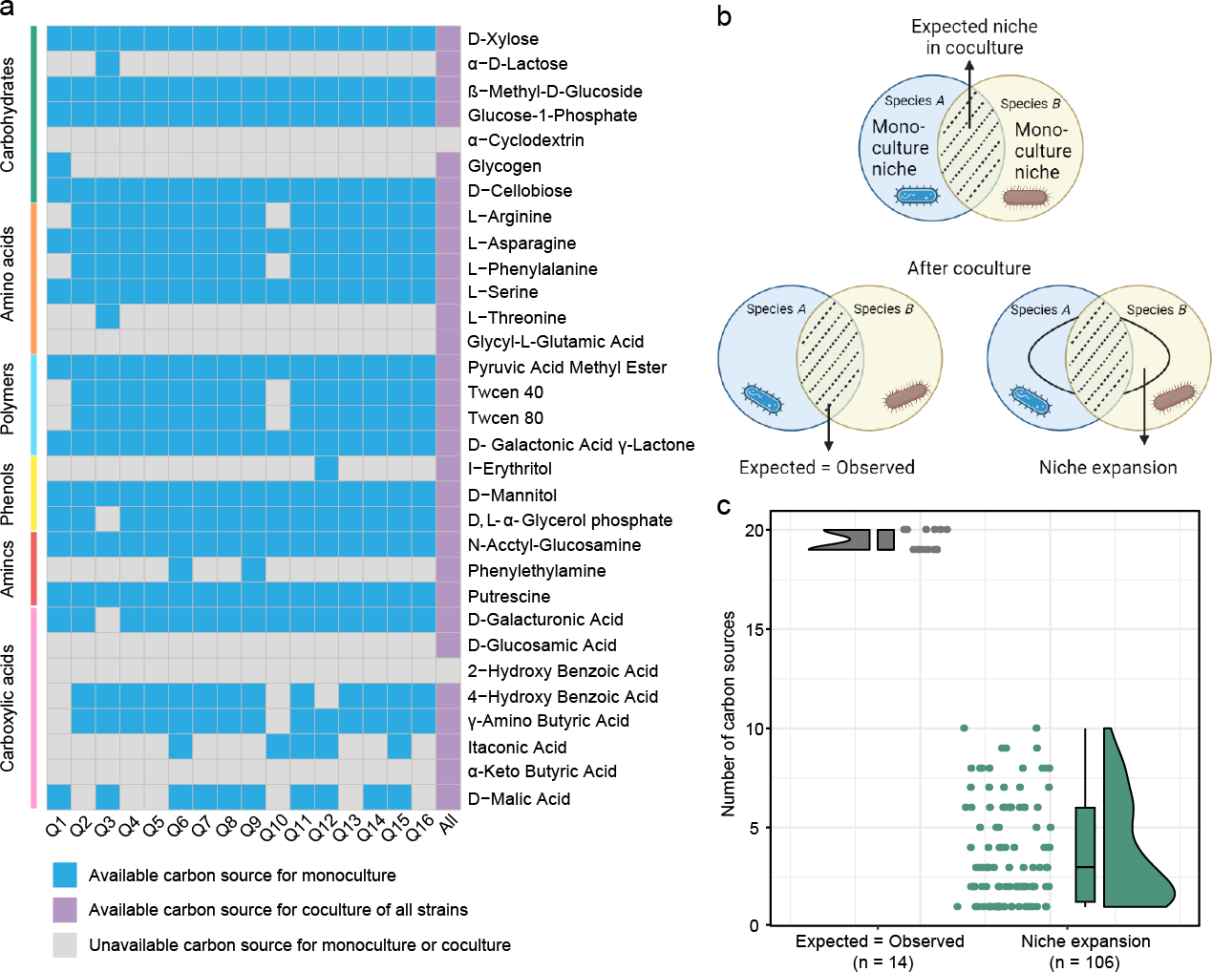
Pairwise interactions influence niche deformation between bacterial species. (a) A schematic matrix capturing resources between the given bacteria (blue boxes) and the synthetic community of all of them (All, purple boxes). Colored squares indicate that the given bacteria consume a given resource. (b) Deformation of niche space in bacterial interactions. Circles represent the set of carbon sources where each partner can grow (i.e., its metabolic niche). The intersection between sets represents the carbon sources where both partners are predicted to grow in co-culture. Observed growth in co-culture is represented by the black-shaded set. Two kinds of niche deformation may occur after co-culture: “Expected = Observed,” indicating a perfect match between the expected number of carbon sources used in monoculture and the growth observed in co-culture, and “Niche expansion,” signifying that the niche space occupied by the co-culture is larger than the set expected from combined monocultures. (c) The degree of overlap in the number of carbon sources utilized by the two monocultures (expectation) and co-cultures (observation) was quantified following a 7-day growth period. Data points represent the number of carbon sources in which a particular combination of genotypes grew as expected (gray) or could use more (green, niche expansion) carbon sources than predicted based on the growth pattern of the two monocultures.

We developed a pairwise synthetic community composed of two different genera from the 16 bacterial strains isolated from the watermelon rhizosphere (Table 1). We first studied the individual niche space (i.e., the number of carbon sources utilized) of each strain and the niche space of the community synthesized by all strains. We observed distinct niches for each strain, and the niche space of the synthetic community, comprising all strains, significantly expanded compared to that of each individual strain. Notably, certain carbon sources, previously unavailable to all individual strains, were now available (Fig. 2a). To explore the relationship between the interaction of synthetic community members and niche deformation, we determined the trend of niche deformation through a paired Biolog co-culture experiment (Fig. 2b) (Table S1). Here, “Expected = Observed” represents the number of carbon sources in which a particular combination of strains grew as expected. The term “niche expansion” was defined as the condition in which none or only one partner could utilize a certain carbon source under the monoculture of the two strains, but both strains could use the C source under pairwise co-culture. By comparing the expected carbon utilization profile with the carbon utilization actually observed in experiments, it was determined that the growth pattern of monocultures accurately predicted the observed pattern in 11.7% of cases (a total of 14 out of 120 pairs) (Fig. 2c). Interestingly, about 88.3% (a total of 106 out of 120 pairs) of cases exhibited different degrees of niche expansion in co-cultures because of potential synergy (Fig. 2c). Notably, when niche expansion occurs, the number of carbon sources in which a co-culture can grow increases by three on average compared to the expected, and the number of pairs with niche expansion was considerably higher than the number of pairs in the “Expected = Observed” group, suggesting that there may be more cooperative relationships between the bacterial species.

**TABLE 1:** Identification based on 16S rRNA gene sequencing of some bacteria isolated from the rhizosphere of grafted watermelon plants^*a*^

Original identification number	Identifier in this study	Closest phylogenetic affiliation in the GenBank/EMBL/DDBJ databases	Similarity (%)	Accession no.
JSACC60222	Q1	*Pseudoxanthomonas mexicana*	97.2	MZ242608.1
JSACC60215	Q2	*Pseudorhodoferax aquiterrae*	99.3	NR108842.1
JSACC60115	Q3	*Rhizobium daejeonense*	100	AY341343.1
JSACC60160	Q4	*Lysobacter panacisoli*	96.21	MK850379.1
JSACC60213	Q5	*Sphingopyxis soli*	99.92	NR116739.1
JSACC60214	Q6	*Pseudomonas azotifigens*	98.41	KE384407.1
JSACC60225	Q7	*Ensifer numidicus*	98.88	FJ919756.1
JSACC60228	Q8	*Nocardioides kongjuensis*	99.85	NR043651.1
JSACC60229	Q9	*Microbacterium arborescens*	100	CP128474.1
JSACC60220	Q10	*Enterobacter bugandensis*	97.88	KI911561.1
JSACC60217	Q11	*Achromobacter mucicolens*	96.61	NR117613.1
JSACC60218	Q12	*Olivibacter jilunii*	99.85	NR109321.1
JSACC60223	Q13	*Aeromonas media*	100	JN829160.1
JSACC60230	Q14	*Bosea eneae*	99.69	ON138901.1
JSACC60231	Q15	*Arthrobacter* sp.	94.17	CP014196.1
JSACC60224	Q16	*Acinetobacter pittii*	96.66	AP024415.1

^*a*^ The sixteen bacteria isolated from grafted watermelon rhizosphere.

### Metabolic similarity influences niche deformation between bacterial species

**Fig 3:**
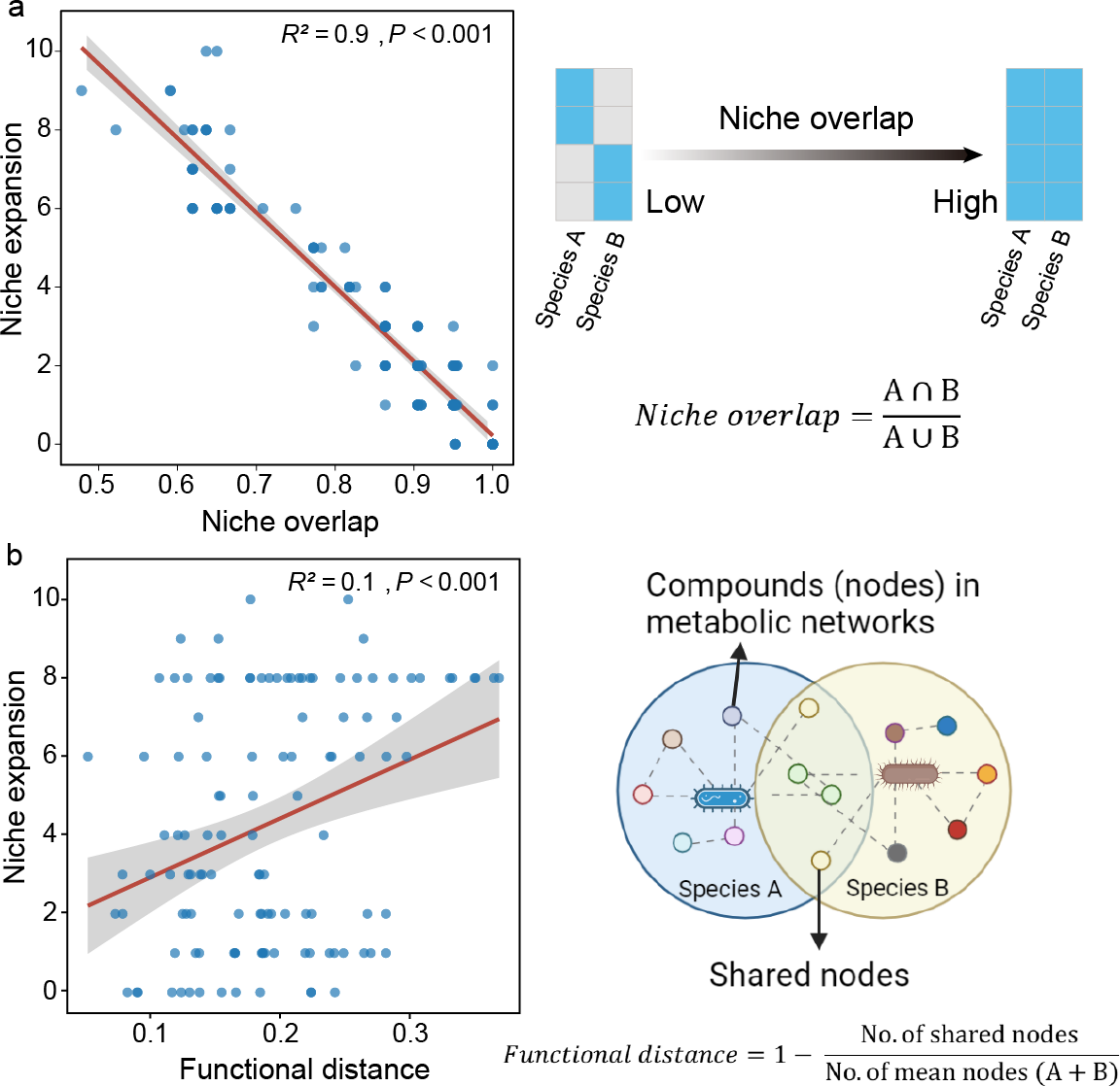
Niche overlap and functional distance drive niche expansion. (a) The relationship between niche expansion and the niche overlap index (*n* = 120). The description of niche overlap index is presented on the right. (b) The relationship between niche expansion and functional distance (*n* = 120). The description of functional distance is presented on the right.

To identify factors that shape the niche spaces of pairwise co-cultures, we examined whether metabolic similarity (niche overlap and functional distance) of monoculture could be used to predict niche expansion in all co-cultures (*n* = 120). We found that niche expansion was negatively correlated with niche overlap index (Pearson correlation, *P* < 0.001, *R*^2^ = 0.9, *n* = 120, Fig. 3a), and niche expansion was positively correlated with functional distance (Pearson correlation, *P* < 0.001, *R*^2^ = 0.1, *n* = 120, Fig. 3b). These results demonstrate that the increased frequency of niche expansion is attributed to microbial pairwise interactions, and the overlap of niche and metabolic networks may represent a potential driving mechanism.

### Resource availability drives changes in interactions through competition for resources

**Fig 4:**
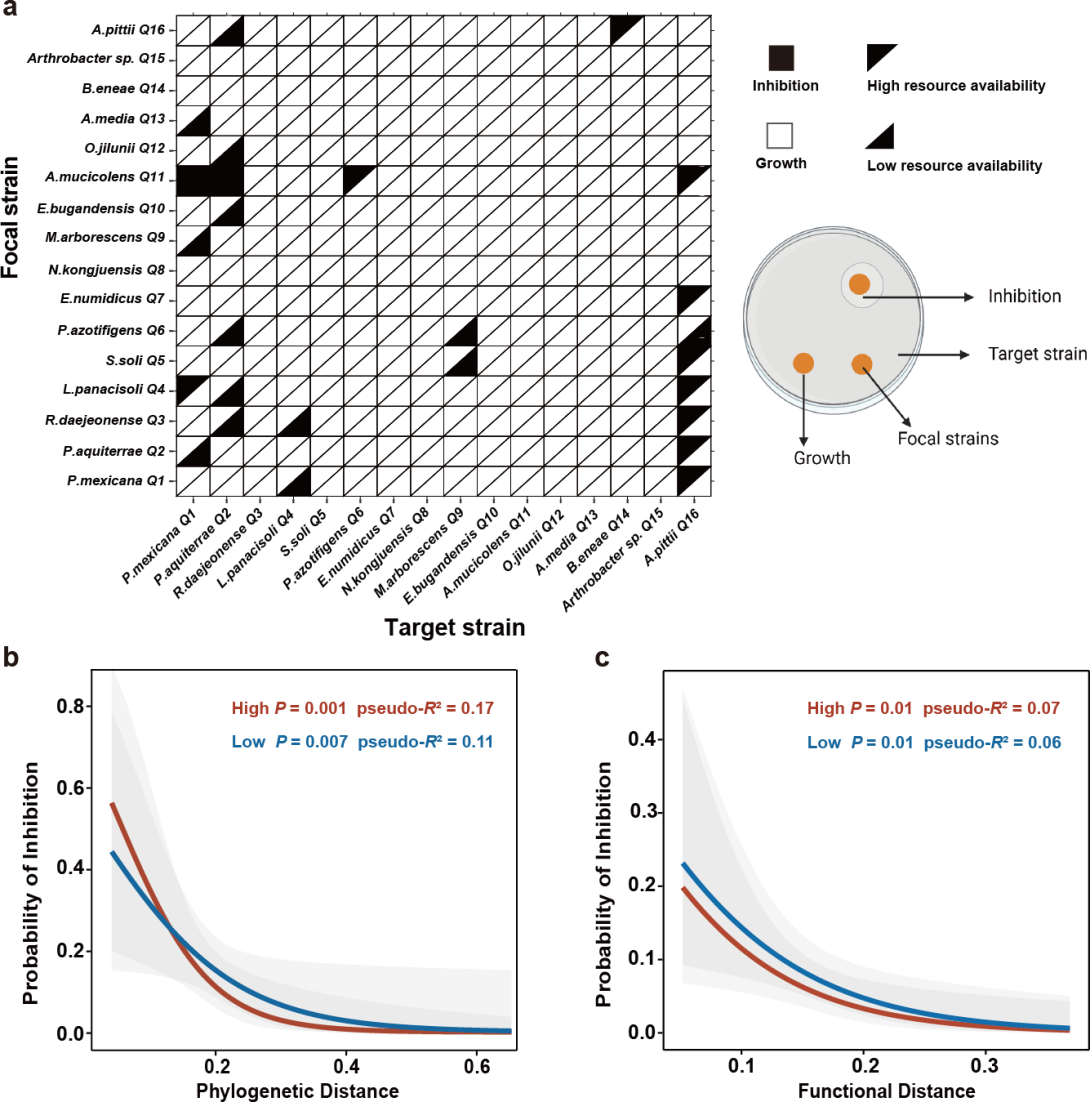
Altered inhibition during co-culture under high and low resource availabilities. (a) Inhibition matrix of pairwise cultivation at low and high resource availabilities. Triangles indicate whether a target strain showed normal growth (white) or was inhibited (black) by the focal strain. Each square is divided into upper and lower triangles, where the upper triangle represents the result under high resource availability, and the lower triangle represents the data under low resource availability. (b) Logistic regression between phylogenetic distance and the probability of inhibition (*P*_High_ = 0.001, pseudo-*R*^2^ = 0.17, *n* = 120; *P*_Low_ = 0.007, pseudo-*R*^2^ = 0.11, *n* = 120). (c) Logistic regression between functional distance and the probability of inhibition (*P*_High_ = 0.01, pseudo-*R*^2^ = 0.07, *n* = 120; *P*_Low_ = 0.01, pseudo-*R*^2^ = 0.06, *n* = 120). The red and blue lines represent the logistic regression curves, and the gray shaded area indicates the 95% confidence intervals.

As observed in our assays, pairwise interactions in synthetic communities could significantly expand niches between bacterial species, and niche overlap and functional distance between bacterial species were highlighted as a potential driving mechanism. Given that these overlaps or similarities may involve competition for resources, we further investigated the impact of resource availability on pairwise interactions of co-cultures of these strains. We next focused on the influence of resource limitation on bacterial interactions and explored the driving forces and mechanisms responsible for interactional changes. We observed that 28 pairs of all pairwise interactions (120 pairs at high and low concentrations, respectively) were inhibitory, with 12 pairs (10.0%) versus 16 pairs (13.3%) inhibitory at high and low resource availabilities, respectively (Fig. 4a). This suggested that the frequency of inhibition was marginally higher under low resource conditions (McNemar’s *χ*^2^ = 0.38, df = 1, one-tail *P* = 0.27; Fig. 4a). Furthermore, the probability of bacterial inhibition is greater when resource availability is low (Fig. 4b). We identified a point of intersection between high and low resource availabilities in the logistic regression curve of phylogenetic distance and inhibition (Fig. 4b), which may be due to differences in phylogenetic traits that encompass differences in fitness as well as niches between strains.

Among all the inhibiting bacterial pairs, we found that inhibition was strongest between phylogenetically closely related strains at both high and low resource availabilities (logistic regression, *P*_High_ = 0.001, pseudo-*R*^2^ = 0.17, *n* = 120; *P*_Low_ = 0.007, pseudo-*R*^2^ = 0.11, *n* = 120; Fig. 4b). To assess whether the association between inhibition and phylogenetic distance is driven by the metabolic network overlap between the strains, we fitted the functional distance and inhibition effect and found a clear negative correlation between the two at both resource availability (logistic regression, *P*_High_ = 0.01, pseudo-*R*^2^ = 0.07, *n* = 120; *P*_Low_ = 0.01, pseudo-*R*^2^ = 0.06, *n* = 120; Fig. 4c). Together, these results suggest that metabolic similarity (metabolic network overlap) promotes the competition of strains for a specific resource. This result agrees with the identified relationship between functional distance and niche expansion (Fig. 3b).

### Mechanisms by which resource availability modulates bacterial pairwise interactions

**Fig 5:**
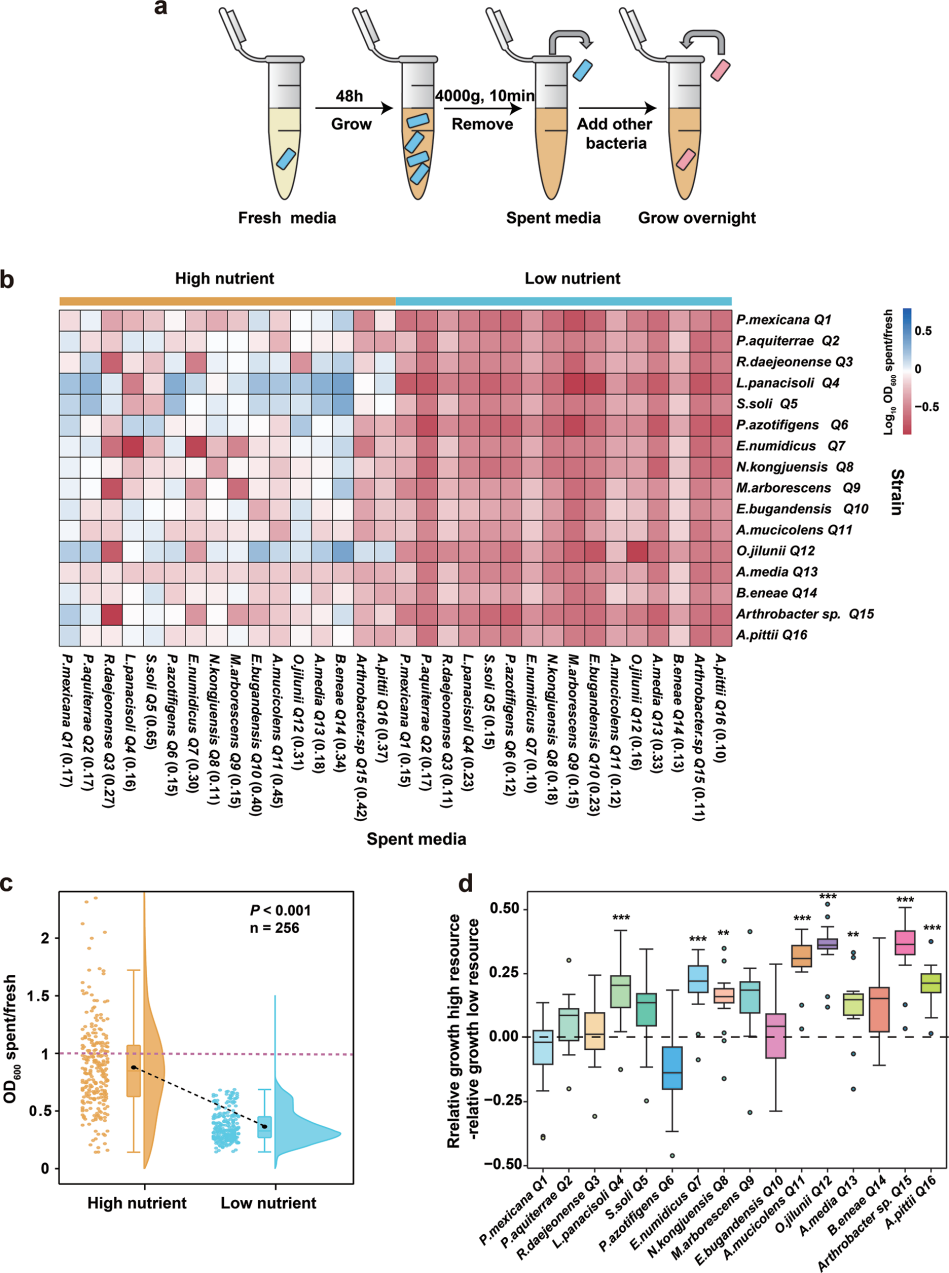
Effects of resource availability on bacterial interactions. (a) Bacteria were cultured under high and low resource availabilities, respectively. The spent media were obtained by removing the bacteria, and then other strains were added to re-grow. (b) Heatmap denoting the ratio of OD_600_ in the spent media to OD_600_ in the fresh media for pairwise co-cultures at high and low resource availabilities (log_10_ transformed). Values significantly greater than 0 indicate positive interactions, and values significantly less than 0 indicate negative interactions. The numbers in parentheses in the horizontal coordinates are the growth rates (h^−1^) of each strain under high and low resource availabilities, respectively. (c) Box plot showing significant differences in bacterial growth in spent medium between high and low resource availabilities (ANOVA followed by Dunnett’s T3 *post hoc* test, *P* < 0.001, *F*_(1,510)_= 2056, *n* = 256). (d) Plot showing differences in the relative growth of bacteria under high and low resource availabilities. Values above 0 indicate relative facilitation growth. Values below 0 indicate a stronger inhibition of the interaction partner. We tested whether each group of values is significantly greater than 0. Asterisks indicate statistically significant differences (***P < 0.001 and **P < 0.01) based on analysis of variance with Dunnett’s T3 *post hoc* test.

Niche space deformations are the outcome of bacterial interactions. To assess the effects of resource competition caused by resource availability on interactions between species in the synthetic communities, we compared the growth and interaction of each bacterial strain in the presence of supernatant from each of the other bacteria. The supernatant was collected from high (100% NB) and low (10% NB) media (see Materials and Methods), respectively (Fig. 5a). We observed that strains that grew in spent media at high resource availability typically exhibited lower growth, though this growth was not completely inhibited (Fig. 5b). Positive interactions were still identified in 81 out of 256 cases (each case contained three replications, and 81 cases were significantly greater than 0, *P* < 0.05; Fig. 5b). In contrast, strains that grew in spent media at low resource availability exhibited complete growth inhibition. Significant differences in strain growth at high and low resource conditions in spent media were observed (ANOVA followed by a Dunnett’s T3 *post hoc* test, *P* < 0.001, *F*_(1,510)_= 2056, *n* = 256; Fig. 5c). Finally, we tested differences in the relative growth of bacteria under high and low resource availabilities. Here, the values above 0 indicated relative facilitation growth. High resource availability resulted in more facilitation (>0) in 71% of cases (each case contained three replications, and 182 cases were significantly greater than 0, *P* < 0.05; Fig. 5d). The results suggest that resource availability significantly influenced the interactions of bacterial pairwise consortia, including the effective induction of strong negative interactions under low resource availability. In conclusion, our supernatant assay indicated that these negative interactions are mainly driven by resource competition via the uptake and production of many different inhibitory metabolites.

## DISCUSSION

The interaction of species in microbial communities has long been a focus in ecological research (1, 3, 20, 21). Here, we explored how metabolic similarity and resource availability affect niche change by influencing microbial interactions. We showed that metabolic similarities (functional distance and niche overlap) between interacting partners are key factors in determining niche deformation in interactions between different bacteria. Given that these metabolic similarities may involve competition for resources, we further assessed the effects of the degree of resource competition on direct competition or cooperative interactions between species in synthetic communities. We observed a widespread facilitation among bacteria in environments with high resource availability, highlighting the possibility of expanding ecological niches under such conditions. Conversely, under the low resource availability condition, bacteria tend to inhibit one another because of heightened competition, limiting the potential for niche expansion. Our study has broad implications for understanding the significance of resource availability in determining microbial interactions as well as for facilitating the design of synthetic microbial communities. This knowledge will ultimately enable targeted microbiome design, which is pivotal to microbiome applications in health, agriculture, and the environment.

Here, we found that the niche of the synthetic community tends to be more extensive than that of individual strains, encompassing even the carbon sources inaccessible to all individual strains (Fig. 2a). This may be because cooperation between bacteria in a synthetic community can effectively promote the growth of complementary partners, such as cross-feeding (8), or disinhibition of growth (26), thereby activating the ability to use other new carbon sources and stabilizing the entire consortium. In our pairwise synthetic communities, niche expansion is more prevalent than niche “Expected = Observed” in our synthetic communities (Fig. 2c), indicating that interactions between strains tend to be more cooperative over a wide range of C sources. A possible mechanism for niche expansion is that, in addition to the individual species niche that is manifested in co-cultures, cross-feeding between strains of secondary metabolites may allow for the growth of partners and niche expansion (8). Alternatively, two strains might produce extracellular enzymes, converting the original carbon sources into available resources for other species and fostering niche expansion (27). Notably, we observed that niche overlap, determined by carbon resource utilization patterns, strongly correlated with niche expansion (Fig. 3a). To illustrate this niche deformation mechanism, we introduced metabolic distance (i.e., metabolic network overlap) (Fig. 3b). Niche deformation via microbial resource competition networks was previously observed in bacterial communities (28). Our study extends this understanding to diverse bacterial combinations. Two metabolically similar strains exhibit a greater overlap in their metabolic networks and niche spaces, resulting in greater resource competition and reducing the amount of niche expansion. This is basically consistent with our hypothesis. Overall, niche deformation varies based on the differences in resource consumption preferences (metabolic network) of the interacting strains and the properties of bacterial dissimilarity (niche overlap).

In line with recent studies (29, 30), we found that strong competitive interactions between bacteria in resource-limited conditions may not be driven by the production of toxic metabolites but by competition for resources, possibly because limited resources do not allow bacteria to produce large amounts of inhibitory secondary metabolites. We thus suggest that the niche deformation in our study was mainly driven by resource competition due to the overlap of niche and metabolic networks. To further confirm that resource availability (degree of resource competition) alters bacterial pairwise interactions with effects on niche deformation, we set up an antagonism assay and a supernatant assay under high and low resource availability conditions. Our results show that in all pairwise interactions, strains grown under low resource availability had a marginally higher probability of antagonism than those grown under high resource availability (Fig. 4a) (11, 31, 32). This may be because the trade-off between microbial cooperation and competition depends largely on environmental conditions (10), such as changes in resource supply (33). If the benefits of cooperation are lower relative to the costs of competition, this leads to more inhibition between species. Some interacting species have been shown to fiercely compete for resources in the face of limited public goods (34) under low resource conditions. High resource conditions (external resource supplemented) represent a high metabolite flux and the adequacy of resources between interacting species in a community. This high resource availability results in species utilizing each other’s metabolites to achieve cooperation or to promote growth (33). If the interacting species benefit from cross-feeding more than the cost of resource competition, they will have a weaker antagonistic tendency. As expected, with the increase of functional distances and phylogenetic distance (when phylogenetic distance is larger than the point of intersection), the probability of inhibition was higher under low resource availability than under high resource availability (Fig. 4b and c). Based on these results, as a general rule, it appears that inhibition or competition may be the best response to resource stress.

Remarkably, we also noticed that the logistic regression curves of high and low resource availabilities have a point of intersection in terms of the phylogenetic distance (Fig. 4b). The phylogenetic distance can be represented by both fitness and niche differences between species (35, 36). A shorter phylogenetic distance is probably more related to the fitness difference, and a longer phylogenetic distance is more related to the niche difference (36). Thus, the fact that the phylogenetic distance is smaller than this intersection can be explained by fitness differences. Bacteria with shorter phylogenetic distances have similar fitness and resource assimilation capabilities, enabling rapid growth and larger populations under high resource availability. This inevitably increases the competition between species for the same required resources (37) and thus leads to higher inhibition (Fig. 4b). As the phylogenetic distance increases over this intersection, the effect of niche differences should override the effect of fitness differences. Distantly related species have larger niche differences and smaller niche overlap. Each takes what it needs and competes less strongly with other species under high resource conditions. However, under low resource conditions, each species competes for these limited and same required resources, leading to increased inhibition (38, 39).

To clarify the mechanisms by which resource availability modulates bacterial pairwise interactions, we performed a fermentation supernatant (spent media) assay to test the prevalence of positive and negative interactions and their variations in all pairwise co-cultures at both high and low resource availabilities (Fig. 5a). We observed that spent media were able to support the growth of other species, especially at high resource availability (Fig. 5b). Based on the growth rates of these strains under conditions of different nutrient concentrations (Fig. S2 and S3), we ruled out any possibility that low growth was hidden under low nutrient conditions. Especially under resource-rich conditions, cooperative interactions, such as cross-feeding, can enhance metabolic efficiency (8, 40, 41), leading to significant bacterial growth (Fig. 5c). Meanwhile, low resource availability resulted in a higher prevalence of negative interactions (Fig. 5d). This could be attributed to the scarcity of resources, leading bacteria to reduce the utilization and excretion of metabolites, thereby intensifying food scarcity and competition (19, 39). These competitive interactions vary with resource pressure and bioavailability. Competition is maximal and rates of dilution (i.e., low residence times or high diffusion rate of resources) should be low when resources are limited. Bacterial access to resources depends largely on diffusive transport in the aqueous phase, which in turn is affected by resource availability (42). Therefore, the decrease in the number of bacterial niche expansions is due to the negative effects of resource competition (Fig. S1). When resources are plentiful, dilution rates are high, and bacteria produce more metabolites. This improves the overall resource utilization rate of newly added bacteria and facilitates more rapid microbial growth. Under such conditions, positive interactions dominate, enhancing the potential of bacteria to exploit new ecological niches (17, 43). These findings align with our hypothesis: the fewer metabolic similarities between species, the less competition for resources they cause and the more they tend to cooperate, promoting niche expansion. Although our study is based on pairwise co-cultures, it offers a stepwise approach to move from a simple synthetic community to a complex, multi-component community.

In conclusion, we systematically investigated the effects of metabolic similarity and nutritional status on niche deformation and interactions among members of synthetic communities. Our results suggest that metabolic similarity with bacteria is a mechanism driving observed niche deformation. Metabolic similarity may involve different degrees of resource competition, which can change pairwise interactions within the synthetic community and thus potentially modulate the bacterial community niche. Our research is essential to comprehensively understand microbial interactions and their effects on individual niches, helping to stabilize communities in the face of fluctuating resource availability. Our results provide direct experimental evidence elucidating both cooperative and competitive interactions among bacteria in numerous pairwise co-cultures under different nutrient statuses. These findings highlight the critical influence of nutrient status in balancing cooperation and competition within bacterial communities. Here, we uncovered several general, statistical rules governing microbial community structure and function. We will explore whether our findings apply to additional phylogenetic groups in follow-up studies, aiming to generalize the trends observed in our system.

## MATERIALS AND METHODS

### Bacterial strains and growth conditions

The 16 bacterial strains used in this study (Table 1) were isolated from the rhizosphere soil of watermelon plants using NB medium (see Supplementary Methods). All bacteria were streaked to a single colony to confirm their purity. To prepare pre-cultures, glycerol stocks frozen at −80°C were streaked on NB (10 g/L bacto tryptone, 5 g/L NaCl, and 5 g/L beef extract) with 2% agar plates and grown for 24 h at 30°C. Then, a single colony was picked and cultured in NB medium, and the initial pH was adjusted to 7.0. All optical density (OD) measurements were performed with a SpectraMax i3x microplate reader at 600 nm. In all the experiments, three independent biological replicates were used.

### Niche space quantification and profiling via carbon sources utilization

The niche space was evaluated by quantifying the catabolic activities of monocultures and co-cultures of strains. The Biolog EcoPlates (Biolog, Hayward, USA) were used to detect the carbon (C) source utilization of each strain individually or in co-cultivation in three replicates. The Biolog contained 31 different C sources. Water without any C source served as a control in triplicates. The Biolog system distinguished and identified bacteria based on the differences in the utilization of C sources by strains. The inoculation methods of Biolog were as follows. To prepare the culture to be transferred into the Biolog, 200 µL of pre-cultures of bacteria (equal OD_600_) were inoculated into 20 mL of phosphate-buffered saline (PBS). In the co-cultures, two pairwise strains or all strains (co-culture of 16 strains) were mixed with equal volume and equal OD_600_, and each strain was inoculated with 100 or 12.5 µL of the diluted precultured bacteria, respectively. Finally, each well of the Biolog was inoculated by 140 µL of culture, and plates were sealed with a transparent film. To determine the growth and C source utilization pattern and number, the absorbance (595 nm) was measured at 0, 4, and 7 days after inoculation, respectively. All treatments included co-cultures (*n* = 120) and monocultures (*n* = 16), which were replicated three times independently in this experiment. All Biolog plates were incubated at 30°C and 170 r.p.m.

#### Phenotypic profiles and mathematical definitions of ecological niche properties

To analyze the data from the Biolog cultivation experiment, negative measurements were set to zero to avoid mathematical errors when subtracting values from each other. The absorbance of the water control (blank control) was subtracted for each well to correct for basal growth in all treatments. Afterward, to correct for the initial absorbance of C sources and the initial cell density, the measurements of the starting day (day 0) were subtracted from other time points (day 4 and day 7). The growth of the strain in the specific tested C source was scored as positive if the absorbance, after subtracting the blank control and the starting day (day 0), was above the 97.5% percentile of the blank values (equivalent to a 5% significance level for two-tailed distributions, *P* < 0.05). For all treatments and controls, the mean growth value of three independent replicates was used for further analysis (8) (Table S1).

We took 31 carbon sources that bacteria can grow on as a set of vectors defining phenotypic profiles. For example, the phenotypic profiles of two partners A and B were represented by their vectors of the form *A* = (1, 0, 0, …, 1) and *B* = (0, 1, 0, …, 1), where each vector size was *n* = 31. Therefore, niche space was defined as the number of C sources in which a strain could grow (8).

Niche expected was simply the intersection between vectors:


Niche expected=A∩B

The observed niche, that is, the number of carbon sources that the two strains could utilize in co-culture, was defined as *O* as a vector set.

Niche expansion was defined as follows:


Niche expansion ={(A∪B) − (A∩B)} ∩ O

That is, the condition in which none or only one partner could utilize a certain carbon source under the monoculture of the two strains, but both strains could use the C source under pairwise co-culture.

Niche overlap was defined as follows:


$$Niche\;overlap\;=\frac{A\cap B}{A\cup B}$$

### Phylogenetic distance and functional distance analysis

To construct the phylogenetic tree for the 16 different species, full-length 16S ribosomal RNA gene sequences (~1.5 kb) were blasted against NCBI GenBank. The tree was constructed using the neighbor-joining method in MEGA X software (44) using the Tajima-Nei method. The phylogenetic distances between species were derived from the resulting distance matrix.

To generate the functional distance of the different species, the 16S rRNA gene sequences were first blasted against the Greengenes v13.5 16S rRNA gene database (45) with prfectBLAST (46). The best hit was selected from the Kyoto Encyclopedia of Genes and Genomes (KEGG) Ortholog table provided by PICRUSt2 that matches the 16S rRNA gene (47). Next, the metabolic networks were generated from the list of KEGG Orthologs with RevEcoR (threshold = 0, is giant = FALSE) (48). Functional similarity was then calculated for each pair as the overlap between nodes (metabolites) in the two networks divided by the mean number of nodes in both networks (11). The functional distance was then defined as one subtracted by the functional similarity. Here, A and B stood for species


$$Functional\;distance\;=1-\;\frac{No.of\;shared\;nodes}{No.of\;mean\;nodes\;\left(A\;+\;B\right)}$$

### Antagonism assay

A screening of all possible pairwise interactions was performed using the sterile plate assay to test for inhibition between the selected strains. To address the role of resource limitation in inhibition, we tested whether inhibition changed if the C source concentration was reduced by 10-fold. Therefore, two C resource concentrations were set to be high (normal NB) and low (10% NB), respectively. Broth cultures of the 16 strains were grown in high and low resource concentrations at 30°C for 24 h and were each spread onto three high and low resource square plates to create bacterial lawns (defined as target strain) (aliquots of 200 µL). Next, 16 Oxford cups (diameter: 0.5 cm) were put on each plate, and aliquots (100 µL) of each bacterial broth culture were transferred into separate cups (defined as focal strain). Focal strains were tested for their capacity to inhibit each target strain. All 16 strains were tested as both focal and target strains, leading to 512 possible assays (16 strains × 16 strains × 2 resources), with 32 of those being self-inhibition controls. Inhibition was detected as a zone of clearance surrounding the colony after 3 days of incubation (18). For each pair of strains, inhibition was defined and valued as follows: if A inhibited B, *x* = 1; if B inhibited A, *y* = 1; both 0 if no inhibition was observed. We denoted pairs to be antagonistic if *x* and/or *y* were 1. Logistic regression (glm, binomial family) was used to test for associations between inhibition, phylogenetic distance, and functional distance. Here, the independent variable was the phylogenetic distance and functional distance between strains in each pair co-culture, and the dependent variable was a binary variable indicating whether there was inhibition between strains (1/0). Every interaction was assessed at least in triplicate.

### Estimation of growth rate

The 16 isolates were grown in high and low resource conditions (NB broth) at 30°C for 2 days, respectively. A Bioscreen C Microbiological Growth Curves Analysis System (Labsystems, Helsinki, Finland) was used to serially assess turbidity as a measurement of growth. Data were recorded, and OD_600_ was recorded every 30 min. For each strain (five replicates), a logistic growth curve was fitted, and the growth rate (h^−1^) was calculated with the R package “growthcurver” (49).

### Supernatant assay

A new pre-culture was set up in high and low resource conditions, as described above. Both bacteria were grown for 48 h at 30°C (by measuring the growth curves of each strain, we found that OD_600 nm_ saturated for each species in monoculture after 48 h, indicating that the original nutrients had been consumed). The cell cultures were spun down (10 min; 4,000 *g*), and the supernatant was filtered through sterile filters (0.22 µm) with a 50 mL sterile syringe. As described above, the filtered supernatant was defined as spent media. To verify sterility, 100 µL of this spent media was plated on lysogeny broth agar plates. At the same time, the bacteria were grown at 20 mL high and low resource overnight at 30°C, respectively. The bacteria were spun down (10 min; 4,000 *g*) and re-suspended in 20 mL of PBS. Before the supernatant assay, all cell concentrations were adjusted to equal OD_600 nm_ using PBS. Those bacteria were then diluted 1/100× into the corresponding spent media and into the corresponding fresh media described above. These cultures were grown at 30°C in 96 deepwell plates (200 µL per well) and shaken at 170 r.p.m. To test how environmental changes affected bacterial interactions through cross-feeding, the OD_600_ of the cultures in the different resource spent media was measured and divided by the OD_600 nm_ of the corresponding species cultured in a fresh medium after overnight culturing. The resulting data are shown in Fig. 5b. The color intensity represents the ratio (log_10_ transformed) in the heatmap. Three independent biological replicates were used.

### Statistical analysis

All data analyses were performed in R 4.0.2. Part of the data was visualized online using ImageGP (50). Statistical differences between groups were inferred using ANOVA followed by Dunnett’s T3 *post hoc* test. Statistical associations between variables were inferred using the Pearson correlation test.

## Data Availability

Additional information is provided in the supplementary material (Appendix S1), and Biolog experimental raw data are provided in Table S1 (Niche Raw Data). The strains were deposited into the Jiangsu Agricultural Microbial Germplasm Resources Collection, as detailed in the original identification number in Table 1.

## SUPPLEMENTAL MATERIAL

Supplemental figures aem.01566-23-s0001.docx Supplemental text and Fig. S1 to S3.

Experiment raw data aem.01566-23-s0002.xlsx Niche experiment raw data.

ASM does not own the copyrights to Supplemental Material that may be linked to, or accessed through, an article. The authors have granted ASM a non-exclusive, world-wide license to publish the Supplemental Material files. Please contact the corresponding author directly for reuse.
