# Supplementary material for: Nutrient status changes bacterial interactions in a synthetic community
Source: Appl Environ Microbiol. 2023 Dec 21;90(1):e01566-23. doi: 10.1128/aem.01566-23 (PMC10807438; doi:10.1128/aem.01566-23)
Supplement: Supplemental figures — Supplemental text and Fig. S1 to S3. [file aem.01566-23-s0001.docx]

**APPENDIX S1 for**

**Nutrient status changes bacterial interactions in a synthetic community**

Yizhu Qiao ^a^, Qiwei Huang ^a, *^, Hanyue Guo ^a^, Meijie Qi ^a^, He Zhang ^a^, Qicheng Xu ^a, b^, Qirong Shen ^a^, Ning Ling ^b,^

a, Key lab of organic-based fertilizers of China and Jiangsu provincial key lab for solid organic waste utilization, Nanjing Agricultural University, Nanjing 210095, China

b, Centre for Grassland Microbiome, State Key Laboratory of Grassland Agro-Ecosystems, College of Pastoral Agriculture Science and Technology, Lanzhou University, Lanzhou 730020, China

Corresponding authors:

* Qiwei Huang, Email: [qwhuang@njau.edu.cn](mailto:qwhuang@njau.edu.cn)

**Supplementary Methods**

**Isolation and identification of culturable bacteria**

The grafted watermelon rhizosphere samples from the field were used for the isolation of bacteria. Then rhizosphere samples were suspended in PBS buffer (pH 7.0) and used for bacterial isolation by dilution in culture media. The suspensions were diluted into different concentrations, and about 100 μl dilution was plated in bacterial culture mediums, Beef extract peptone (NB). The single colonies were picked based on the size, color, and morphology. Colonies were re-streaked at least twice to ensure purity. And then these selected single colonies were subsequently inoculated into 2 ml sterile tubes containing the corresponding medium and incubated for 5 days. Afterward, the bacterial DNA was extracted from each isolate using lysis buffer (25 mM NaOH and 0.2 mM Na_2_-EDTA, pH 12) and incubated for 30 min at 95 °C, then neutralizing with the neutralization buffer (40 mM Tris-HCl, pH 7.5) (1). Single colonies were identified based on the full-length 16S rRNA gene sequence (2). Briefly, the full-length of 16S rRNA was amplified with forward primer 27 (5’-AGAGTTTGATCMTGGCTCAG-3’) and primer 1492R (5’- TACGGYTACCTTGTTACGACTTC -3’) (2) under the condition as follows: denaturation at 95° for 4 min, followed by 30 cycles of 95 °C for 30 s, 55 °C for 30 s, and 72 °C for 60 s, and final elongation at 72 °C for 5 min (2). Sanger sequenced was performed at LC-Bio Technology Co., Ltd (Shanghai, China). Sequence alignment was performed on the NCBI website, and each microbial species was preserved in 30% glycerol (v/v) at −80 °C. We utilized a synthetic community composed of 16 bacterial strains isolated from the rhizosphere soil of grafted watermelon plants, which has been demonstrated to promote the growth of own-rooted watermelon plants in our other pot experiment. And some of these strains have been used for interaction studies before (3, 4, 5). This community, in its defined chemical environment, represents a tractable model system for exploring how abiotic and biotic interactions shape the ecological dynamics of microbial communities.

**References**

1. Zhang J, Liu Y, Guo X, Qin Y, Garrido-Oter R, Schulze-Lefert P, Bai Y. 2021. High-throughput cultivation and identification of bacteria from the plant root microbiota. Nature Protocols 16: 988-1012.
2. Li Z, Bai X, Jiao S, Li Y, Li P, Yang Y, Wei G. 2021. A simplified synthetic community rescues Astragalus mongholicus from root rot disease by activating plant-induced systemic resistance. Microbiome 9: 1-20.
3. Oña L, Giri S, Avermann N, Kreienbaum M, Thormann KM, Kost C. 2021. Obligate cross-feeding expands the metabolic niche of bacteria. Nature Ecology Evolution 5: 1224-1232.
4. Ratzke C, Barrere J, Gore J. 2020. Strength of species interactions determines biodiversity and stability in microbial communities. Nature ecology evolution 4: 376-383.
5. Piccardi P, Vessman B, Mitri S. 2019. Toxicity drives facilitation between 4 bacterial species. Proceedings of the National Academy of Sciences 116: 15979-15984.


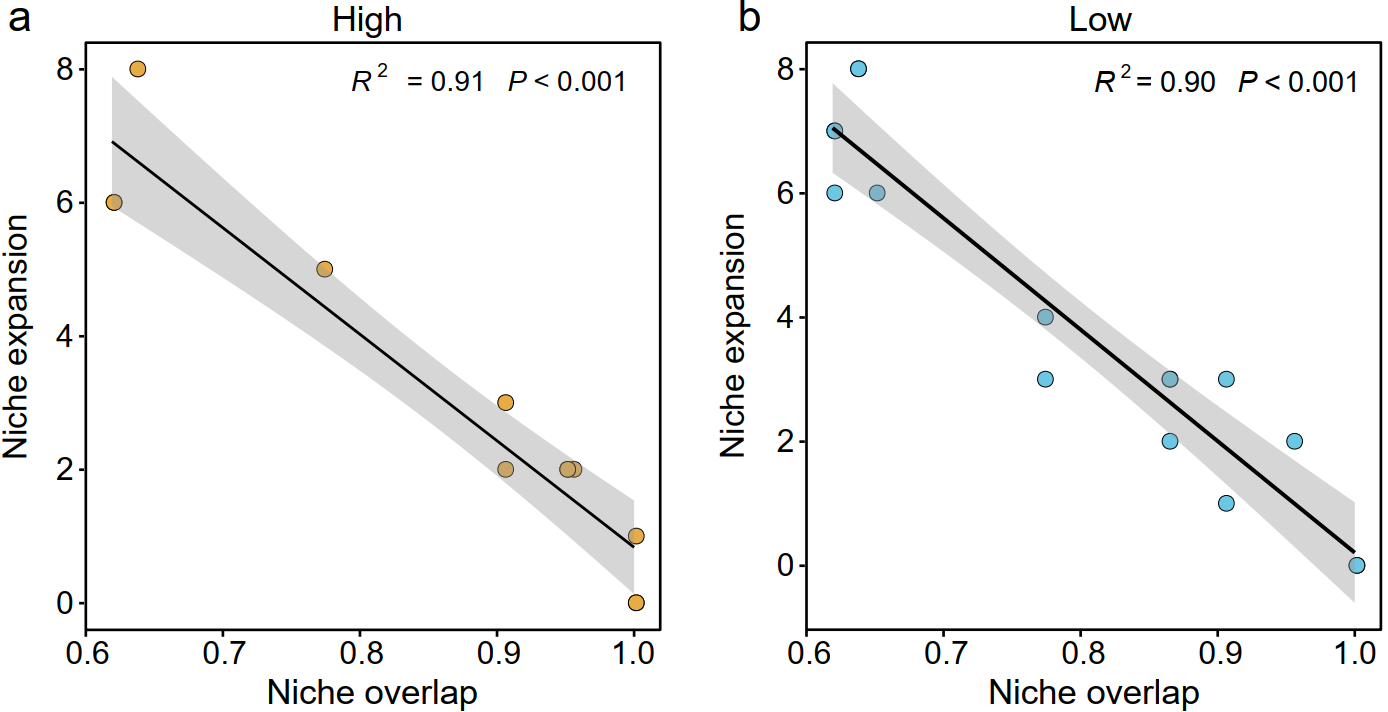


**Figure S1.** Relationship between niche overlap and niche expansion number between pairs of strains with inhibitive relationships. (a) Under the condition of high resource availability (*R*^2^=0.91, *P* < 0.001, n=12); (b) Under the condition of low resource availability (*R*^2^=0.90, *P* < 0.001, n=16). The results show that strains with inhibitive relationships reduce niche expansion due to niche overlap.


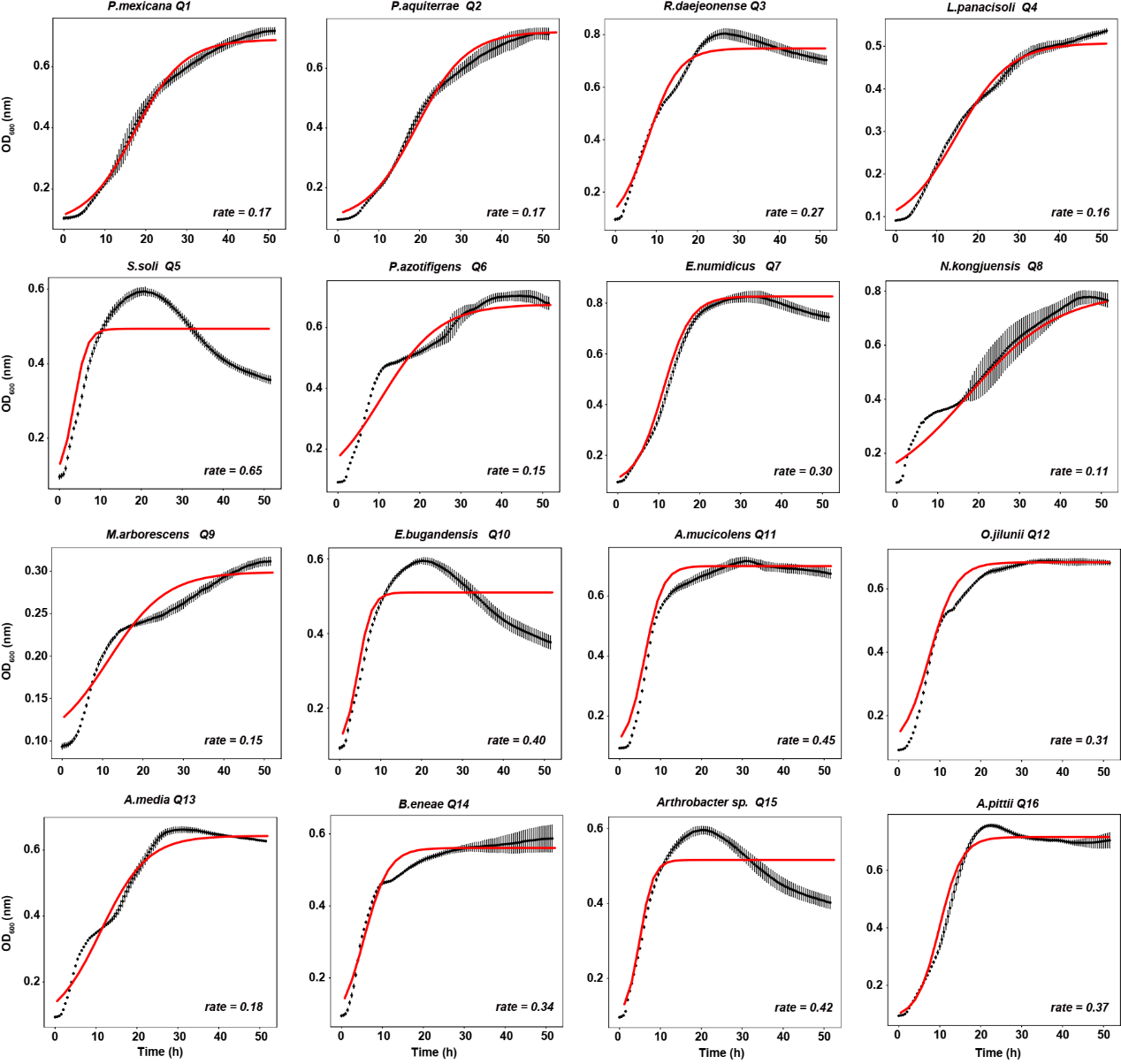
**Figure S2.** Growth curve of each strain under high nutrient condition. Points are means and error bars are standard deviations (5 replicates).


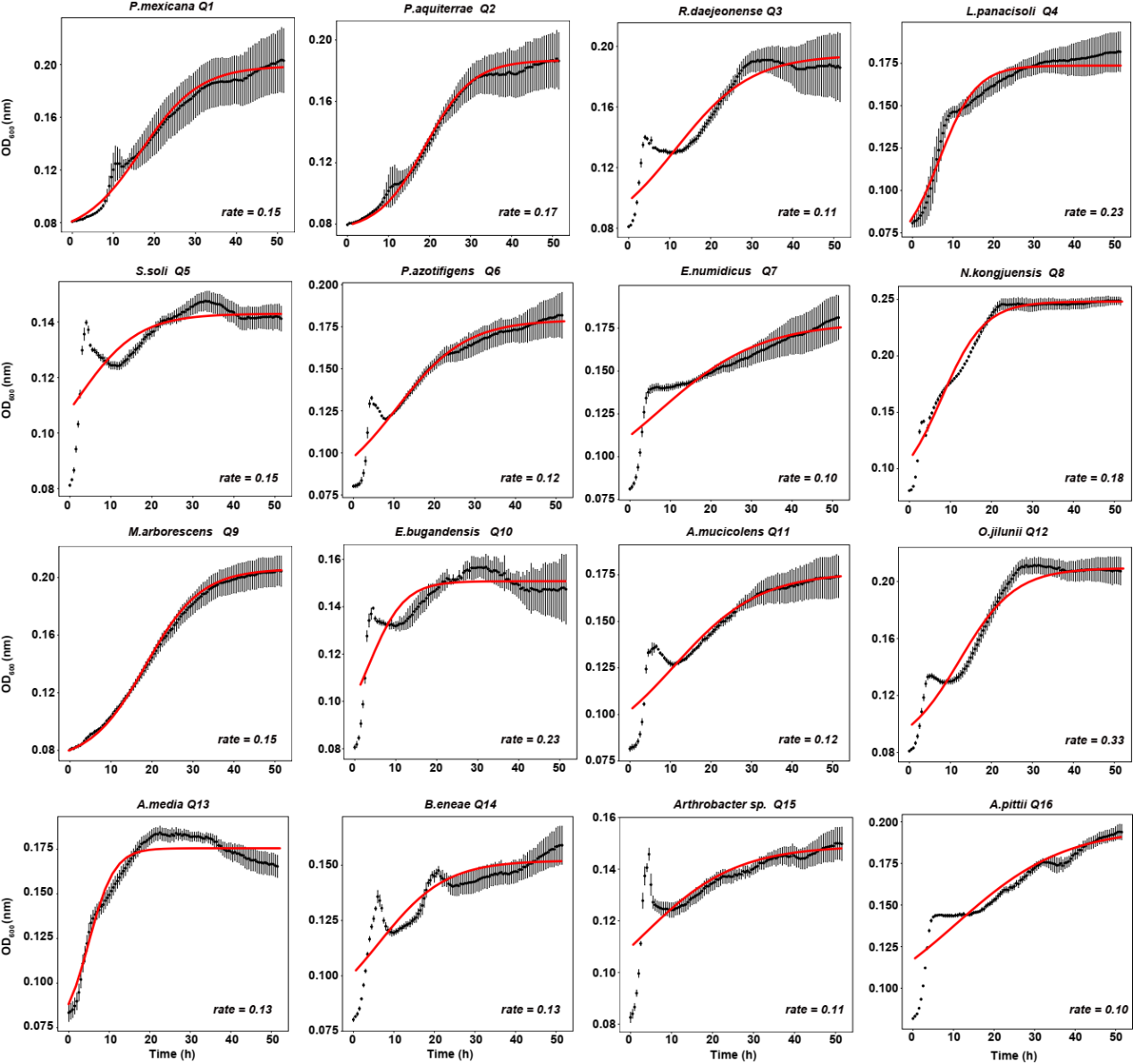
**Figure S3.** Growth curve of each strain under low nutrient condition. Points are means and error bars are standard deviations (5 replicates).
